# Diagnosis of ventilator-associated pneumonia: a systematic review of the literature

**DOI:** 10.1186/cc6877

**Published:** 2008-04-21

**Authors:** Alvaro Rea-Neto, Nazah Cherif M Youssef, Fabio Tuche, Frank Brunkhorst, V Marco Ranieri, Konrad Reinhart, Yasser Sakr

**Affiliations:** 1 Department of Anesthesiology and Intensive Care, Friedrich-Schiller-University Hospital, 07743 Jena, Germany; 2 Department of Anesthesiology and Intensive Care, S. Giovanni Battista Hospital, University of Turin, Turin, 10126, Italy

## Abstract

**Introduction:**

Early, accurate diagnosis is fundamental in the management of patients with ventilator-associated pneumonia (VAP). The aim of this qualitative review was to compare various criteria of diagnosing VAP in the intensive care unit (ICU) with a special emphasis on the value of clinical diagnosis, microbiological culture techniques, and biomarkers of host response.

**Methods:**

A MEDLINE search was performed using the keyword 'ventilator associated pneumonia' AND 'diagnosis'. Our search was limited to human studies published between January 1966 and June 2007. Only studies of at least 25 adult patients were included. Predefined variables were collected, including year of publication, study design (prospective/retrospective), number of patients included, and disease group.

**Results:**

Of 572 articles fulfilling the initial search criteria, 159 articles were chosen for detailed review of the full text. A total of 64 articles fulfilled the inclusion criteria and were included in our review. Clinical criteria, used in combination, may be helpful in diagnosing VAP, however, the considerable inter-observer variability and the moderate performance should be taken in account. Bacteriologic data do not increase the accuracy of diagnosis as compared to clinical diagnosis. Quantitative cultures obtained by different methods seem to be rather equivalent in diagnosing VAP. Blood cultures are relatively insensitive to diagnose pneumonia. The rapid availability of cytological data, including inflammatory cells and Gram stains, may be useful in initial therapeutic decisions in patients with suspected VAP. C-reactive protein, procalcitonin, and soluble triggering receptor expressed on myeloid cells are promising biomarkers in diagnosing VAP.

**Conclusion:**

An integrated approach should be followed in diagnosing and treating patients with VAP, including early antibiotic therapy and subsequent rectification according to clinical response and results of bacteriologic cultures.

## Introduction

Ventilator-associated pneumonia (VAP) is common in the intensive care unit (ICU), affecting 8 to 20% of ICU patients and up to 27% of mechanically ventilated patients [1]. Several risk factors have been reported to be associated with VAP, including the duration of mechanical ventilation, and the presence of chronic pulmonary disease, sepsis, acute respiratory distress syndrome (ARDS), neurological disease, trauma, prior use of antibiotics, and red cell transfusions [2]. Mortality rates in patients with VAP range from 20 to 50% and may reach more than 70% when the infection is caused by multi-resistant and invasive pathogens [1-3]. The incidence of VAP-attributable mortality is difficult to quantify due to the possible confounding effect of associated conditions, but VAP is thought to increase the mortality of the underlying disease by about 30% [3]. VAP is also associated with considerable morbidity, including prolonged ICU length of stay, prolonged mechanical ventilation, and increased costs of hospitalization [3,4].

Delayed diagnosis and subsequent delay in initiating appropriate therapy may be associated with worse outcomes in patients with VAP [1,5,6]; on the other hand, an incorrect diagnosis may lead to unnecessary treatment and subsequent complications related to therapy [1,7,8]. Early, accurate diagnosis is, therefore, fundamental in the management of patients with VAP [9]. Several criteria have been proposed for diagnosing VAP in clinical settings, including clinical manifestations, imaging techniques, methods to obtain and interpret bronchoalveolar specimens, and biomarkers of host response. Due to the lack of an acceptable gold standard, the accuracy of these methods in diagnosing VAP is controversial.

The aim of this qualitative review was, therefore, to compare various criteria for diagnosing VAP in the ICU with a special emphasis on the value of clinical diagnosis, microbiological culture techniques, and biomarkers of host response.

## Materials and methods

We performed a MEDLINE search using the keywords 'ventilator associated pneumonia' AND 'diagnosis'. Our search was limited to human studies published between January 1966 and June 2007. The abstracts of all articles were used to confirm our target population (patients with VAP) and the corresponding full-text articles were reviewed for the presence of data comparing a diagnostic test to a 'gold-standard'. Only studies of at least 25 adult patients were included. Two investigators (AR and NC) independently identified the eligible literature. Predefined variables were collected, including year of publication, study design (prospective/retrospective), number of patients included, and disease group. Any inconsistencies between the two investigators in interpretation of data were resolved by consensus. To avoid publication bias, abstracts and full articles were eligible. We also reviewed the bibliographies of available studies for other potentially eligible studies. Of 572 articles fulfilling the initial search criteria, 159 articles were chosen for detailed review of the full text. A total of 64 articles fulfilled the inclusion criteria and were included in our review.

## Results

### Accuracy of the clinical diagnosis of VAP

There is no single clinical manifestation that can be used alone to diagnose VAP. Chest radiology, although very sensitive, is typically nonspecific [10,11]. Wunderink *et al*. [12] showed that no roentgenographic sign correlates well with pneumonia in mechanically ventilated patients. Lobar or subsegmental atelectasia, ARDS, alveolar hemorrhage, and/or infarction may be mistaken for pneumonia [12]. Other clinical signs (fever, leukocytosis or pulmonary manifestations) have intermediate predictive values [11,13]. The clinical diagnosis of VAP has, therefore, traditionally been made by the association of a new or progressive consolidation on chest radiology plus at least two of the following variables: fever greater than 38°C, leukocytosis or leukopenia, and purulent secretions. These criteria were proposed by Johanson *et al*. [14] (Table 1), and compared to immediate post-mortem lung biopsies by Fàbregas *et al*. [11]. The sensitivity was only 69% and specificity not better than 75% (accuracy of 72%). An increase (or decrease) in the number of clinical criteria, can increase (or decrease) the specificity, but at the cost of sensitivity. Despite this relatively low accuracy, these criteria were recommended by the American Thoracic Society Consensus Conference on VAP [1].

**Table 1:** Clinical criteria used in diagnosing ventilator-associated pneumonia

Johanson criteria	• Presence of a new or progressive radiographic infiltrate		
	• Plus at least two of three clinical features:		
	- fever > 38°C		
	- leukocyto sis or leukopeni		
	- purulent secretions		
	• Temperature	• Oxygenation (PaO2/FiO2)	• Tracheal secretions (score)
	- 0 point: 36.5–38.4 C	- 0 point: PaO2/FiO2 > 240 or ARDS	-0 point: < 14
	- 1 point: 38.5–38.9	- 2 points: PaO2/FiO2 < 240 and no evidence of ARDS	-1 point: > 14
	- 2 points: < 36 or > 39		-2 points: purulent sputum
Clinical Pulmonary Infection Score (CPIS)	• Blood leukocytes (cells/μL)		• Culture of tracheal aspirate
	- 0 point: 4000–11000		-0 point: minimal or no growth
	- 1 point: < 4000 or > 11000		-1 point: moderate or more growth
	- 2 points: > 500 band forms		-2 points: moderate or greater growth
		• Pulmonary radiography	
		-0 point: no infiltrate	
		- 1 point: diffuse or patchy infiltrates	
		- 2 points: localized infiltrate	
	Total score of > 6 points suggests ventilator-associated pneumonia		
	ARDS = acute respiratory distress syndrome		

Centers for Disease Control and Prevention (CDC)	• Radiology signs	• Clinical signs	
	Two or more serial chest radiographs with at least 1 of the following:	At least 1 of the following:	
	- new or progressi ve and persistent infiltrate	- fever (temperat ure > 38 C)	
	- consolidation	- leukopeni a (< 4000 WBC) or leukocyto sis (> 12000 WBC)	
	- cavitation	- altered mental status, for adults 70 years or older, with no other recognized cause	
	• Microbiological criteria		
	At least one of the following:	Plus at least 2 of the following:-	
	- positive growth in blood culture not related to another source of infection	- new onset of purulent sputum, or change in character of sputum	
	- positive growth in culture or pleural field	- increased respiratory secretions, or increased suctioning requirements	
	- positive quantitati ve culture from bronchoal veolar lavage (> 10^4^) or protected specimen brushing (> 10^3^)	- new-onset or worsening cough, or dyspnea, or tachypnea	
	- five percent or more of cells with intracellul ar bacteria on direct microsco pic examinati on of Gram-stained bronchoal veolar lavage fluid	- rales or bronchial sounds	
	- histopathological evidence of pneumonia	- worsening gas exchange	
		- increased oxygen requirements	

Combinations of various criteria to establish a diagnosis in patients with VAP have been suggested and validated (Table 1). The National Nosocomial Infection Surveillance (NNIS) system was developed in the 1970s by the Centers for Disease Control as a tool to describe the epidemiology of hospital-acquired infections and to produce aggregated rates of infection suitable for inter-hospital comparison, but was never compared to pathological results. The NNIS system was compared to bronchoalveolar lavage (BAL) fluid cultures in 292 trauma patients and had a sensitivity of 84% and a specificity of 69% [15]. More recently, the Clinical Pulmonary Infection Score (CPIS) was proposed by Pugin *et al*. [16], based on six variables (fever, leukocytosis, tracheal aspirates, oxygenation, radiographic infiltrates, and semi-quantitative cultures of tracheal aspirates with Gram stain) [16]. The original description showed a sensitivity of 93% and specificity of 100%, but this study included only 28 patients and the CPIS was compared to quantitative culture of BAL fluid using a 'bacterial index' defined as the sum of the logarithm of all bacterial species recovered, which is not considered an acceptable gold standard for the diagnosis of VAP. Compared to pathological diagnosis, CPIS had a moderate performance with a sensitivity between 72 and 77% and specificity between 42 and 85% [11,17]. Likewise, CPIS was not sufficiently accurate compared to a BAL fluid-established diagnosis with sensitivity between 30 and 89% and specificity between 17 and 80% [17-22] (Table 2). Luyt *et al*. [19] studied 201 mechanically ventilated patients in whom strict bronchoscopic criteria were applied to diagnose or exclude pneumonia. The CPIS assessed at baseline was calculated retrospectively and did not differ significantly for patients with or without VAP. The potential use of CPIS as the sole means to diagnosis VAP was also evaluated in 158 trauma patients [18]. The average CPIS was similar between patients with systemic inflammatory response syndrome (SIRS) (BAL < 10^5 ^colony forming units(CFU)/ml) and those with VAP (BAL > 10^5 ^CFUml) with a sensitivity of 61% and specificity of 43%. In 28 patients with burn injuries, Pham *et al*. [23] found that CPIS had a sensitivity of 30% and specificity of 80% in diagnosing VAP compared to quantitative BAL fluid culture.

**Table 2:** Studies comparing clinical criteria to other diagnostic (Dx) tests

First author	Sample	Dx Tests	Gold standard	Results
Fabregas, 1999, ** [11]	Medical ICU, 25 pts	Johanson criteria, CPIS, TBA(10^5^), PSB (10^3^), pBAL(10^4^), BAL(10^4^)	Pathology + Culture	• Johanson criteria (2 items): sens = 69%, spec = 75%. • Any Johanson criteria: Chest Rx: sens = 92%, spec = 33%; leukocytosis: sens = 77%, spec = 58%; fever: sens = 46%, spec = 42%; purulent secretions: sens = 69%, spec = 42% • CPIS: sens = 77%, spec = 42%. • TBA sens = 69%, spec = 92%. • pBAL sens = 39%, spec = 100%. • BAL sens = 77%, spec = 58%. • PSB sens = 62%, spec = 75%. • QtC added little to clinical diagnostic accuracy
Papazian, 1995, ** [17]	Mixed ICU, 38 pts, consecutive	BBS (10^4^) & mini-BAL (10^3^) & PSB (10^3^) & BAL (10^4^) & CPIS	Pathology + Culture	• CPIS: sens = 72%; spec = 85% • BBS (10^4^): sens = 83%, spec = 80% • mini-BAL (10^3^): sens = 67%, spec = 80% • BAL: sens (10^4^) = 58%, spec = 95% • PSB: sens (10^3^) = 42%, spec = 95% • BBS was more accurate than PSB
Croce, 2006 *, # [18]	Trauma ICU, 158 pts	CPIS (>6)	BAL (10^5^)	• Frequency of VAP: BAL ≥ 10^5 ^= 42%, SIRS = 58% • Average CPIS: VAP = 6.9, SIRS = 6.8 • CPIS > 6: sens = 61%, spec = 43%
Luyt, 2004 *, ** [19]	Mixed ICU, 201 pts	CPIS (>6)	PSB (10^3^) BAL (10^4^)	• CPIS: sens = 89%, spec = 44%, k = 0.33, PPV = 57%, NPV = 84%
Schurink, 2004, ** [20]	Mixed ICU, 99 pts	CPIS	BAL (10^4^)	• Frequency of VAP = 69% • ROC curve for CIPS > 6, 7 and 8 = 0.54, 0.64, 0.64; r = 0.115 • CPIS > 5: sens = 83%, spec = 17% • CIPS >6 or ≤ 6: k 0.16
Fartoukh, 2003, ** [21]	Mixed ICU, 68 pts	CE & PIS (>6)	BAL (10^4^) or PTC (10^3^)	• CE: sens = 50%, spec = 59% • CPIS > 6: sens = 60%, spec = 59% • Adding positive Gram stain to CIPS improves diagnostic accuracy
Miller, 2006, ** [15]	Trauma ICU, 292 pts	NNIS	BAL (10^5^)	• k = 0.73. • Sens = 84%, spec = 69%, PPV = 83%, NPV = 70%
Pham, 2007*, ** [23]	Mixed ICU, 28 burn pts	CPIS	BAL	• CPIS: sens = 30%, spec = 80%, PPV = 70%, NPV = 50%
Pugin, 1991, **, [16]	Surgical ICU, 28 pts,	CPIS & mini-BAL (BI ≥ 5)	BAL (BI ≥ 5)	• CPIS: sens = 93%, spec = 100%, r = 84% (CIPS and mini-BAL), r = 76% (CPIS and BAL) • Mini-BAL: sens = 73%, spec = 96%

CPIS = clinical pulmonary infection score; TBA = tracheobronchial aspirate; PSB = protected specimen brush; pBAL = protected BAL or 'mini-BAL'; BAL = bronchoalveolar lavage; sens = sensitivity; spec = specificity; QtC = quantitative culture; BBS = blind bronchial sampling; SIRS = systemic inflammatory response syndrome; PPV = positive predictive value; NPV = negative predictive value; CE = clinical estimate; NNIS = National nosocomial infection surveillance system; BI = bacterial index; ICU = intensive care unit; *retrospective; **consecutive; ^#^convenient.

A major limitation of the literature validating CPIS for diagnosing VAP is that BAL culture is not a true gold standard [11,13,17,24-28]. In addition, the calculation of CPIS was modified by some authors and different cutoff points were used to diagnose VAP [19,20]. Importantly, the inter-observer agreement in calculating CPIS was found to be poor (kappa = 0.16) [20].

In summary: Clinical manifestations are usually used in combination with other features to diagnose VAP. Chest radiography may be sensitive but is typically nonspecific. NNIS criteria do not seem to be reliable for VAP diagnosis at the bedside. CPIS may be a helpful tool in diagnosing VAP, however, the considerable inter-observer variability and the moderate performance of the CPIS should be taken in account. Further studies are warranted to validate clinical criteria against pathological diagnosis.

### The role of bacteriological data in improving the accuracy of a clinical diagnosis of VAP

Many studies have evaluated the value of bacteriological data in establishing the diagnosis of VAP compared to pathological, clinical, or other bacteriological diagnostic criteria (Tables 2, 3, 4, 5, 6). In a study by Torres *et al*. [26], quantitative cultures were obtained through BAL (bacterial count = 10^4 ^CFU), protected BAL (pBAL) (10^4^), protected specimen brush (PSB) (10^3^) and tracheobronchial aspirate (TBA) (10^5^) and were compared to five different histological and microbiological references [26]. Sensitivities for diagnosis of VAP ranged from 22% to 50% when only histologic reference tests were used, whereas specificity ranged from 45% to 100%. When lung histology of guided or blind specimens and microbiology of lung tissue were combined [11,13,16,24-31], all quantitative diagnostic techniques achieved relatively higher, but still limited, diagnostic yields (sensitivity range 19% to 87%; specificity range 31% to 100%). Fabregas *et al*. [11] also showed that addition of the results of quantitative cultures to clinical criteria (Johanson or CPIS) did not increase their accuracy in diagnosing VAP.

**Table 3:** Studies comparing quantitative cultures with pathology

First author	Sample	Dx Tests	Gold standard	Results
Balthazar, 2001, ** [13]	Mixed ICU, 37 pts	BAL (10^4^) & Gram & cells from BAL	Pathology + Culture	• BAL: sens = 19%, spec = 94%; fever: sens = 50%, spec = 76%; leucocytosis (>10000): sens = 60%, spec = 76%; Gram stain: sens = 85%, spec = 94%; total cell (>400000): sens = 90%, spec = 94%.
Torres, 2000, ** [26]	Medical ICU, 25 pts	TBA (10^5^) & PSB (10^3^) & BAL (10^4^) & pBAL (10^4^)	Pathology + Culture	• TBA: sens = 50%, spec = 67%. • PSB: sens = 67%, spec = 75%. • pBAL: sens = 63%, spec = 83%. • BAL: sens = 83%, spec = 68%.
Fabregas, 1999, ** [11]	MIxed ICU, 25 pts	Johanson & CPIS & TBA (10^5^), PSB (10^3^), pBAL (10^4^) and BAL (10^4^)	Pathology + Culture	• Johanson criteria (2): sens = 69%, spec = 75%. • Any Johanson criteria: Chest Rx: sens = 92%, spec = 33%; leukocytosis: sens = 77%, spec = 58%; fever: sens = 46%, spec = 42%; purulent secretions: sens = 69%, spec = 42%. • CPIS: sens = 77%, spec = 42%. • TBA: sens = 69%, spec = 92%. • pBAL: sens = 39%, spec = 100%. • BAL: sens = 77%, spec = 58%. • PSB: sens = 62%, spec = 75%. • QtC increased little to clinical diagnosis accuracy.
Papazian, 1997, # [29]	Mixed ICU, 28 pts	Gram & ICO	Pathology + Culture	• BBS Gram stain: sens = 56%, spec = 73%. • mini-BAL Gram Stain: sens = 44%, spec = 87%. • BAL Gram stain: sens = 56%, spec = 100%. • BBS ICO (>10%): sens = 56%, spec = 40%. • Mini-BAL ICO (>5%): sens = 67%, spec = 53%. • BAL ICO (>4%): sens = 56%, spec = 40%.
Kirtland, 1997, ** [27]	Mixed ICU, 39 pts	TA &PSB & pPSB & BAL & BAL cells	Pathology + Culture	• TA: sens = 87%, spec = 31%. • pPSB: sens = 30%, spec = 81%. • PSB: sens = 44%, spec = 81%. • BAL: sens = 65%, spec = 63%. • >50% neutrophils in BAL: sens = 100%.
Marquette, 1995, ** [28]	Mixed ICU, 28 pts	TA (10^5 ^& 10^6^) & PSB (10^3^) & BAL (10^4^)	Pathology	• TA (10^5^): sens = 63%, spec = 75%. • TA (10^6^): sens = 50%, spec = 85%. • PSB (10^3^): sens = 57%, spec = 88%. • BAL (10^4^): sens = 47%, spec = 100%. • ICO (any%): sens = 36%, spec = 100%.
Torres, 1996 *, ** [25]	Mixed ICU, 25 pts	ICO (≥ 5%), mini-BAL (10^4^) & BAL (10^4^)	Pathology	• ICO (≥ 5%) compared to mini-BAL: PPV = 75%, NPV = 83%. • ICO (≥ 5%) compared to BAL: PPV = 57%, NPV = 8 3%. • Mini-BAL: sens = 22%, spec = 100%. • BAL: sens = 45%, spec = 55%.
Papazian, 1995, ** [17]	MIxed ICU, 38 pts	BBS (10^4^) & mini-BAL (10^3^) & PSB (10^3^) & BAL (10^4^) & CPIS	Pathology + Culture	• CPIS: sens = 72%, spec = 85%. • BBS (10^4^): sens = 83%, spec = 80%. • mini-BAL (10^3^): sens = 67%, spec = 80%. • BAL (10^4^): sens = 58%, spec = 95%. • PSB (10^3^): sens = 42%, spec = 95%.
Torres, 1994, ** [24]	Mixed ICU, 30 pts	TBA (10^5^) & PSB (10^3^) & BAL (10^4^) & Clinical data	Pathology	• Clinical data: fever: sens = 55%, spec = 58%; purulent secretions: sens = 83%, spec = 33%; Rx infiltrate: sens = 78%, spec = 42%. • Pulmonary biopsy culture (≥10^3^): sens = 40%, spec = 45%. • Quantitative cultures: TBA: sens = 44%, spec = 48%; PSB: sens = 36%, spec = 50%; BAL: sens = 50%, spec = 45%.
Rouby, 1989, ** [30]	Surgical ICU, 59 pts	pBAL	Pathology + culture	• pBAL: sens = 80%, spec = 66% to identify VAP; sens = 73% to identify the microorganism.
Chastre, 1984, ** [10]	Mixed ICU, 26 pts	PSB (10^3^)	Lung culture	• PSB correlated well to lung cultures, especially in the subgroup of patients who received no antibiotics during the week preceding their death.

ICU = intensive care unit; BAL = bronchoalveolar lavage; sens = sensitivity; spec = specificity; TBA = tracheobronchial aspirate; PSB = protected specimen brush; pBAL = protected BAL or 'mini-BAL'; CPIS = clinical pulmonary infection score; QtC = quantitative culture; ICO = intracellular organism; BBS = blind bronchial sampling; TA = tracheal aspiration; PPV = positive predictive value; NPV = negative predictive value; * = retrospective; ** = consecutive; # convenient

**Table 4:** Studies comparing quantitative cultures using various techniques or cutoff points

First author	Sample	Dx tests	Gold standard	Results
Mondi, 2005, # [31]	Trauma ICU, 39 pts	TA (10^4^) & (10^5^)	BAL (10^5^)	• TA (10^4^): sens = 95%, spec = 58%, k = 0.5339 (p < 0.0001). • TA (10^5^): sens = 90%, spec = 68%, k = 0.6384 (p < 0.0001).
Brun-Buisson, 2005, ** [32]	Mixed ICU, 68 pts	TA (score ≥4+) & bPTC (10^3^) & PTC (10^3^)	BAL (10^4 ^or ICO > 2%)	• TA: sens = 77%, spec = 81%. • bPTC: sens = 77%, spec = 97%. • PTC: sens = 77%, spec = 94%.
Davis, 2005, # [42]	Trauma ICU, 155 pts	Gram (BAL)	CDC + BAL (10^5^)	• Gram (BAL): sens = 88% (any organism). • Gram (BAL): sens = 73%, spec = 49%; PPV = 78%, NPV = 42%, accuracy = 65% (Gram-negative). • Gram (BAL): sens = 87%, spec = 59%; PPV = 68%, NPV = 83%, accuracy = 74% (Gram-positive).
Croce, 2004, ** [43]	Trauma ICU, 526 pts	BAL (10^4 ^& 10^5^)	BAL (10^5^) and ClEvol	• BAL (10^5^): sens = 95%, spec = 10%. • BAL (10^4^): sens = 99%, spec = 70%.
Miller, 2003, ** [44]	Trauma ICU, 168 pts	BAL (10^2 ^to 10^4^)	BAL (10^5^)	• BAL (10^4 ^and 10^3^): increased sensitivity = 14%. • BAL (10^2^): increased sensitivity = 16%.
Sirvent, 2003, ** [45]	Mixed ICU, 82 pts	ICO	Mini-BAL (10^3^)	• ICO ≥ 2%: sens = 80%, spec = 82%. • ICO ≥ 2% better than 1, 5, 7 and 10%.
Wu, 2002, ** [35]	Medical ICU, 48 pts	TA (10^5^)	PSB (10^3^) or BAL (10^4^)	• TA and PSB: sens = 91%, spec = 72%; PPV = 75%, NPV = 90%. • TA and BAL: sens = 91%, spec = 75%; PPV = 78%, NPV = 90%.
Duflo, 2001, ** [46]	Mixed ICU, 104 pts	Gram stain (mini-BAL)	Mini-BAL (10^3^)	• Gram stain: sens = 76%, spec = 100%, k = 0.73, concordance = 86%.
Prekates, 1998,*, ** [47]			BAL	• Gram stain: sens = 77%, spec = 87%, PPV = 71%, NPV = 90%.
Bello, 1996, *, ** [48]	ICU, 74 pts, consecutive	Mini-PSB	PSB and BAL	• BAL and PSB: concordance = 92%. • mini-PSB and BAL: concordance = 84%. • mini-PSB and PSB: concordance = 85%.
Pugin, 1991, ** [16]	Surgical ICU, 28 pts,	CPIS & mini-BAL (BI ≥ 5)	BAL (BI ≥ 5)	• CPIS: sens = 93%, spec = 100%, r = 84% (CIPS and mini-BAL), r = 76% (CPIS and BAL). • Mini-BAL: sens = 73%, spec = 96%.
Jourdain, 1995, ** [36]	Mixed ICU, 39 pts	TA (10^3 ^to 10^7^)	PSB (10^3^) and ICO (≥ 5%)	• TA (10^3^): sens = 90%, spec = 26%, accuracy = 47%. • TA (10^4^): sens = 84%, spec = 40, accuracy = 54%. • TA (10^5^): sens = 79%, spec = 66%, accuracy = 70%. • TA (10^6^): sens = 68%, spec = 84%, accuracy = 79%, correlation = 40% (TA and PSB). • TA (10^7^): sens = 21%, spec = 92%, accuracy = 68%.
Marik, 1995, ** [37]	Medical ICU, 53 pts	Mini-PSB (10^3^)	PSB (10^3^)	• Mini-PSB and PSB: quantitative agreement = 85%.
Kollef, 1995, # [38]	Medical ICU, 42 pts	Mini-BAL (10^3^) & PSB (10^3^)	Johanson (ATS)	• Mini-BAL: sens = 100%, spec = 95%. • PSB: sens = 71%, spec = 100%. Good agreement between mini-BAL and PSB cultures: k = 0,63, concordance = 83%.
Rumbak, 1994, # [39]	Mixed ICU, 38 pts	TA	PSB (10^3^)	• TA: sens = 97%, spec = 50%, PPV = 91%, NPV = 80%.
Valles, 1994, ** [49]	Mixed ICU, 42 pts	ICO & BAL	Clinical criteria + PSB (10^3^)	• BAL (10^3^): sens = 89%, spec = 79%, PPV = 76%, NPV = 90%. • BAL (10^4^): sens = 89%, spec = 100%, PPV = 100%, NPV = 92%. • ICO (≥ 2%): = 78%, spec = 88%, PPV = 82%, NPV = 84%. • ICO (≥ 5%): = 67%, spec = 96%, PPV = 92%, NPV = 79%. • ICO (≥ 7%): = 67%, spec = 100%, PPV = 100, NPV = 80%. • ICO for Pseudomonas: lower sens. • Previous antibiotic treatment decreased sens.
El-Ebiary, 1993, ** [40]	Medical ICU, 102 pts	TA, PSB and BAL	Clinical ad hoc	• TA (10^5^): sens = 70%, spec = 72%, accuracy = 71%. • PSB (10^3^): sens = 60%, spec = 93, accuracy = 64%. • BAL (10^4^): sens = 57%, spec = 87%, accuracy = 67%.
Elatrous, 2004, ** [33]	Medical ICU, 100 pts,	TA (10^2 ^to 10^6^)	PTC (10^6^)	• TA (10^2^): sens = 96%, spec = 66%. • TA (10^3^): sens = 94%, spec = 66%. • TA (10^4^): sens = 92%, spec = 85%, k = 0,78. • TA (10^5^): sens = 84%, spec = 90%. • TA (10^6^): sens = 44%, spec = 94%.
Mimoz, 2000, ** [50]	Mixed ICU, 134 pts,	Gram stain (10 and 50 fields)	PSB (10^3^) or bPTC (10^3^)	• Gram (10 fields) vs PSB: sens = 74%, spec = 94%. • Gram (10 fields) vs PTC: sens = 81%, spec = 100%. • PSB: correlation between morphology and culture: sens = 54%, spec = 86%. • bPTC: correlation between morphology and culture: sens = 69% and spec = 89%.
Flanagan, 2000, ** [22]	Mixed ICU, 145 pts,	Mini-BAL (104) & BAL (104) & PSB (103) & CPIS & BI (≥5)	Clinical (modified CDC)	• Mini-BAL: sens = 74%, spec = 70%, PPV = 17%, NPV = 96%. • BAL: sens = 76%, spec = 71%, PPV = 35%, NPV = 93%. • PSB: sens = 68%, spec = 86%, PPV = 54%, NPV = 95%. • CPIS > 7: sens = 85%, spec = 91%, PPV = 61%, NPV = 96%. • BI: sens = 62%, spec = 53%.
Allaouchiche, 1999, ** [51]	Mixed ICU, 118 pts,	ICO (≥ 2%) & Gram stain (BAL)	PSB (10^3^)	• ICO: sens = 86%, spec = 78%, PPV = 68%, NPV = 91%, k = 0.616, concordance = 81.5%. • Gram stain: sens = 90%, spec = 73%, PPV = 64%, NPV = 91%, k = 0.58, concordance = 79.4%. • Correlation between morphology and culture: complete = 51%, partial = 39.2%, no correlation = 9.8%.
Casetta, 1999, ** [53]	Mixed ICU (Cancer), 42 pts	PTC (10^3^)	PSB (10^3^)	• PTC: sens = 67%, spec = 93%; PPV = 71%, NPV = 91%, agreement = 87%.
Souweine, 1998, ** [54]	Mixed ICU, 52 pts	Antibiotic use & ICO (≥ 5%), PSB (10^3^) & BAL (10^5^)	Clin ad hoc	• ICO: sens = 71% (no antibiotics), 50% (current antibiotics), 67% (recent antibiotics). • PSB: sens = 88% (no antibiotics), 77% (current antibiotics), 40% (recent antibiotics). • BAL: sens = 71% (no antibiotics), 83% (current antibiotics), 38% (recent antibiotics).
Allaouchiche, 1996, ** [52]	Mixed ICU, 132 pts	ICO	PSB (10^3^) + clinical evolution	• ICO (≥ 2%): sens = 84%, spec = 80%, PPV = 69%, NPV = 90%, ROC = 0.888.
Barreiro, 1996, ** [55]	Mixed ICU, 93 pts	pBAL (10^4^) & Gram stain & ICO (≥ 2%)	PSB (10^3^) + Follow-up	• pBAL: sens = 87%, spec = 91%, PPV = 87%, NPV = 91%. • ICO: sens = 75%, spec = 98%, PPV = 96%, NPV = 86%.
Torres, 1989, ** [41]	Mixed ICU, 34 pts	BAL & BI	PTC & BI	• BAL: spec = 71%, r = 0,72 (between BAL and BI) • PTC: spec = 86%, r = 0.78 (between PTC and BI)
Timsit, 1993, ** [56]	Mixed ICU, 26 pts	PSB 1	PSB 2 (2 minutes interval)	• PSB 1: sens = 67%, spec = 94%. • PSB 2: sens = 54%, spec = 94%.
Gerbeaux, 1998, ** [57]	Mixed ICU, 44 pts	BAL 1	BAL 2 (30 minutes interval)	• BAL 1-BAL2 repeatability = 75% with no bias, agreement = 47%.
Butler, 2004, ** [58]	Surgical ICU, 34 pts	Blind PSB	Directed PSB	• Blind PSB and Directed PSB: concordance = 53%.
Wood, 2003, ** [34]	Trauma ICU, 90 pts	TA & BDPB & BPB & BAL	BDPB & BAL	• TA & BAL: k = 0.535. • BPB & BDPB: k = 0.467. • BPB & BAL: k = 0.547.

ICU = intensive care unit; TA = tracheal aspiration; BAL = bronchoalveolar lavage; sens = sensitivity; spec = specificity; bPTC = blinded protected telescoping catheter; PTC = protected (plugged) telescoping catheter; ICO = intracellular organism; CDC = center for disease control; PPV = positive predictive value; NPV = negative predictive value; ClEvol: clinical evolution; PSB = protected specimen brush; CPIS = clinical pulmonary infection score; BI = bacterial index; ATS = American Toracic Society; vs = versus; BDPB = bronchoscope-directed protected brushings; BPB = blind protected brushing via endotracheal tube; *retrospective; **consecutive; ^#^convenient.

**Table 5:** Studies comparing quantitative cultures to clinical diagnosis

First author	Sample	Dx tests	Gold standard	Results
Camargo, 2004, ** [59]	Mixed ICU, 106 pts	TA (10^5 ^and 10^6^) & TA (qualit)	Clinical (ad hoc)	• TA (10^6^): sens = 26%, spec = 78%, PPV = 20%, NPV = 83% • TA (10^5^): sens = 65%, spec = 48%, PPV = 21%, NPV = 87% • TA (qualit): sens = 81%, spec = 23%, PPV = 18%, NPV = 86%
Mentec, 2004, ** [60]	Mixed ICU, 63 pts	TA (10^5^), bPTC (10^3^), PTC (10^3^), BAL (10^4^)	Clinical + Rx (ad hoc)	• For quantitative cultures: TA: sens = 82%, spec = 67%, ROC = 0,78; bPTC: sens = 62%, spec = 94%, ROC = 0,83; PTC: sens = 71%, spec = 94%, ROC = 0,85; BAL: sens = 94%, spec = 100%, ROC = 0.98 • For Gram stain: TA: sens = 94%, spec = 50%; bPTC: sens = 56%, spec = 94%; PTC sens = 65%, spec = 83%; BAL sens = 100%, spec = 94%
Woske, 2001, ** [61]	Surgical ICU, 103 pts, consecutive	BAL (10^4^) & PSB (10^3^) & TA (10^5 ^and 10^6^)	CPIS ≥6	• BAL: sens = 90%. • PSB: sens = 83%. • TA (10^5^): sens = 90%. • TA (10^6^): sens = 50%.
Meduri, 1992, ** [84]	Mixed ICU, 25 pts	pBAL (10^4^) & PSB	Clinical	• pBAL: Spec = 100%, NPV = 100%
Salata, 1987, ** [85]	Mixed ICU, 51 pts	TA	Clinical	• TA: higher Gram stain grading for neutrophils: p < 0.05 • TA: higher bacterial colony count: p < 0.05
Castro, 1991, ** [86]	Mixed ICU, 103 pts	PSB (10^3^)	Clinical	• PSB: sens = 84%, spec = 67%

ICU = intensive care unit; TA = tracheal aspiration; qualit. = qualitative; BAL = bronchoalveolar lavage; sens = sensitivity; spec = specificity; bPTC = blinded protected telescoping catheter; PTC = protected (plugged) telescoping catheter; PPV = positive predictive value; NPV = negative predictive value; PSB = protected specimen brush; CPIS = clinical pulmonary infection score; pBAL = protected bronchoalveolar lavage; **consecutive

**Table 6:** Studies comparing cytological examination to quantitative cultures

First author	Sample	Dx tests	Gold standard	Results
Davis, 2005, # [42]	Trauma ICU, 155 pts	Gram (BAL)	CDC + BAL (10^5^)	• Gram (BAL): sens = 88% (any organism). • Gram (BAL): sens = 73%, spec = 49%, PPV = 78%, NPV = 42%, accuracy = 65% (Gram-negative). • Gram (BAL): sens = 87%, spec = 59%, PPV = 68%, NPV = 83%, accuracy = 74% (Gram-positive).
Sirvent, 2003, ** [45]	Mixed ICU, 82 pts	ICO	Mini-BAL (10^3^)	• ICO ≥ 2%: sens = 80%, spec = 82%. • ICO ≥ 2% better than 1, 5, 7 and 10%.
Duflo, 2001, ** [46]	Mixed ICU, 104 pts	Gram stain (mini-BAL)	Mini-BAL (10^3^)	• Gram stain: sens = 76%, spec = 100%, k = 0.73, concordance = 86%.
Mimoz, 2000, ** [50]	Mixed ICU, 134 pts	Gram stain (10 and 50 fields)	PSB (10^3^) or bPTC (10^3^)	• Gram stain (10 fields) vs PSB: sens = 74%, spec = 94%. • Gram stain (10 fields) vs PTC: sens = 81%, spec = 100%. • Gram (50 fields): slight increase in spec, decrease in sens • Morphology and PSB culture: sens = 54%, spec = 86%. • Morpholgy and bPTC culture: sens = 69%, spec = 89%.
Allaouchiche, 1999, ** [51]	Mixed ICU, 118 pts	ICO (≥ 2%) & Gram stain (BAL)	PSB (10^3^)	• ICO: sens = 86%, spec = 78%, PPV = 68%, NPV = 91%, k = 0.616, concordance 81.5%. • Gram stain: sens = 90%, spec = 73%, PPV = 64%, NPV = 91%; k = 0.58, concordance 79.4%. • Correlation between morphology and culture: complete: 51%, partial: 39.%, no correlation: 9.8%.
Allaouchiche, 1996, ** [52]	Mixed ICU, 132 pts	ICO	PSB (10^3^) + clinical evolution	• ICO (≥ 2%): sens = 84%, spec = 80%, PPV = 69%, NPV = 90%, ROC = 0.888.
Torres, 1996, *, ** [25]	Mixed ICU, 25 pts	ICO (≥ 5%), mini-BAL (10^4^) & BAL (10^4^)	Pathology	• ICO (≥ 5%) compared to mini-BAL: PPV = 75%, NPV = 83%. • ICO (≥ 5%) compared to BAL: PPV = 57%, NPV = 83%. • Mini-BAL: sens = 22%, spec = 100%. • BAL: sens = 45%, spec = 55%.
Sole-Violan, 1994, ** [68]	Mixed ICU, 33 pts	ICO (BAL) & BAL (10^4^) & PSB (10^3^)	Clinical ad hoc	• BAL: sens = 87%, spec = 100%. • PSB: sens = 75%, spec = 100%. • ICO (>4%): sens = 62%, spec = 100%.
Brasel, 2003, ** [66]	Surgical ICU, 35 pts	ICO 5% & ICO 7%	TA (10^4^) & TA (10^5^)	• ICO 5% and TA (10^4^): sens = 61%, spec = 89%, PPV = 90%, NPV = 59%, ROC = 0.84. • ICO 5% and TA (10^5^): sens = 85%, spec = 82%, PPV = 70%, NPV = 91%, ROC = 0.89. • ICO 7% and TA (10^4^): sens = 39%, spec = 97%, PPV = 96%, NPV = 50%, ROC = 0.86. • ICO 7% and TA (10^5^): sens = 61%, spec = 91%, PPV = 77%, NPV = 82%, ROC = 0.84.
Timsit, 2001, ** [65]	Mixed ICU, 110 pts	BAL-D (1% of infected cells)	BAL (10^4^) & PSB (10^3^)	• BAL-D: sens = 93%, spec = 91%, AUC = 0.953, PPV = 90%, NPV = 98%.
Prekates, 1998, ** [47]	Surgical and Trauma ICU, 75 pts,	Gram stain (BAL)	BAL	• Gram stain: sens = 77%, spec = 87%, PPV = 71%, NPV = 90%.

ICU = intensive care unit; BAL = bronchoalveolar lavage; CDC = center of disease control; sens = sensitivity; spec = specificity; PPV = positive predictive value; NPV = negative predictive value; ICO = intracellular organism; PSB = protected specimen brush; bPTC = blinded protected telescoping catheter; vs = versus; PTC = protected (plugged) telescoping catheter; TA = tracheal aspiration; BAL-D = direct examination of BAL; *retrospective; **consecutive; ^#^convenient.

Quantitative cultures obtained by different methods, including BAL, pBAL, PSB or TBA, seem to be rather equivalent in diagnosing VAP [22,31-58] (Table 4). Compared to a pathologically confirmed diagnosis of VAP, BAL (10^4^) had a sensitivity between 19 and 83% and specificity between 45 and 100%, PSB (10^3^) a sensitivity between 36 and 83% and specificity between 50 and 95%, pBAL (10^4^) a sensitivity between 39 and 80% and specificity of 66 to100%, and TBA (10^5^) a sensitivity between 44 to 87% and specificity between 31 to 92% (Table 3) [10,11,13,17,24-30]. Torres *et al*. [26] demonstrated that prior antibiotic use considerably decreased the sensitivity of the cultures of BAL samples. Kirtland *et al*. [27] and Rouby *et al*. [30] reported that the microorganism identified in quantitative culture from a transtracheal method frequently does not correlate well with the culture obtained from pathological samples. In addition, several studies [59-61] have challenged the reliability of quantitative cultures. Timsit *et al*. [56] reported moderate intra-individual variability in comparing two consecutive PSB procedures performed in the same lung. Likewise, Gerbeaux *et al*. [57] performed two BAL procedures in the same lung area to distinguish between the presence and absence of bacterial pneumonia and showed a repeatability of only 75%. Butler *et al*. [58] compared the results of blind PSB sampling from the contralateral lung and PSB from the site of the observed infiltrates; the concordance rate between the two samples was only 53%.

The rate of positive blood cultures in VAP ranges from 8 to 20% [62,63]. In 162 patients with suspected VAP, Luna *et al*. [64] showed that the sensitivity of blood cultures in 90 patients with VAP confirmed by BAL was only 26% and, in many cases, the bacteria isolated in the blood cultures probably had an extrapulmonary source. Blood cultures were also positive in 5 of 72 patients without VAP (6.9%).

Since bacteriological cultures require some days for the results to be available at the bedside, several studies have investigated the value of cytological characteristics of the available bronchoalveolar specimens in diagnosing VAP. These characteristics include the number of inflammatory cells and Gram stains. The rapid availability of cytological data has been shown to be useful in initial therapeutic decisions in patients with suspected VAP [42,46,47,50,51,65-67]. The presence of more than 2% inflammatory cells had a sensitivity of 75% to 86% and a specificity of 78% to 98% in diagnosing the first episode of VAP (Table 6). Other studies [46,49,50,68] investigated other cutoff values and reported contradictory results. Prior antibiotic use was also shown to influence the number of inflammatory cells in patients with VAP [45,54]. Infections with *Pseudomonas aeruginosa *were also shown to decrease the sensitivity of cytological data in diagnosing VAP compared with other bacteria [49]. The presence of bacteria in Gram stains of bronchoalveolar specimens had a sensitivity of 44% to 90% and specificity of 49% to 100% in identifying patients with VAP (Table 6). Davis *et al*. [42] showed that the accuracy of Gram stains was slightly better for Gram-positive than for Gram-negative microorganisms. Although the presence of bacteria on Gram stain appears to have a reasonable accuracy compared to quantitative culture available two to three days later, the agreement between the two methods ranges from 79.4 to 86% (Table 6).

In summary: Bacteriologic data do not increase the accuracy of diagnosis when compared to clinical diagnosis. Quantitative cultures obtained by different methods, including BAL, pBAL, PSB or TBA seem to be fairly equivalent in diagnosing VAP. Blood cultures are relatively insensitive to diagnose pneumonia. The rapid availability of cytological data, including inflammatory cells and Gram stains, may be useful in initial therapeutic decisions in patients with suspected VAP but may be influenced by prior antibiotic use and the infecting microorganism.

### Biomarkers and VAP diagnosis

Many biological markers have been studied in an effort to improve the rapidity and performance of current diagnostic procedures in VAP. When the anatomical and mechanical defense mechanisms that prevent microorganisms from reaching alveoli are overwhelmed, a complex host response develops [1]. Microbial products activate alveolar macrophages, which release multiple endogenous mediators locally. Among these mediators, tumor necrosis factor alpha, interleukin-1β, and other cytokines are increased in various types of pulmonary infections and thus have potential prognostic implications. However, there is no cutoff value for such mediators that could be used to diagnose pneumonia. Several biomarkers have been investigated for diagnosing VAP (Table 7). The presence of elastin fiber (EF), a marker of parenchymal lung destruction, in tracheal secretion has been proposed to differentiate colonization from infection of the lung. However, the presence of EF in tracheal aspirates had low sensitivity (32%) and only reasonable specificity (72%) in diagnosing VAP [69]. In 22 patients with ARDS, Shepherd *et al*. [70] found that EF had a sensitivity of only 40% in diagnosing VAP. This may be due to the fact that EF correlates with lung destruction, more than infection *per se*.

**Table 7:** Studies evaluating the value of biomarkers in diagnosing VAP

First author	Sample	Dx Tests	Gold standard	Results
Povoa, 2005, # [73]	Mixed ICU, 112 pts	CRP	Johanson	CRP (>9.6 mg/dl): sens = 87%, spec = 88%, AUC = 0.92.
Gibot, 2004, ** [75]	Mixed ICU, 148 pts	sTREM-1 in mini-BAL	Mini-BAL (10^3^) Clinical (ad hoc)	Sens = 98%, spec = 90%.
Duflo, 2002, ** [71]	Mixed ICU, 96 pts	PCT serum & alveolar	Mini-BAL (10^3^)	Serum PCT (≥3.9 ng/ml): sens = 41%, spec = 100%, AUC = 0787. Alveolar PCT: not useful.
Oppert, 2002, ** [72]	Mixed ICU, 28 pts	PCT and PCR	Clinical (ad hoc)	Serum PCT (>1 ng/ml): sens = 100%, spec = 75%.
El-Ebiary, 1995, *, ** [69]	Mixed ICU, 78 pts	Elastin fibre	Clinical (ad hoc)	EF: sens = 32%, spec = 72%.
Determann, 2005, ** [76]	Mixed ICU, 28 pts	sTREM-1	Clinical & NBLF	Sens = 75%, spec = 84%.
Pugin, 1992, ** [78]	Trauma ICU, 40 pts	BAL Endotoxin	Clinical & BAL	BAL endotoxin > 6 EU/ml suggests pneumonia due to Gram-negative bacteria.
Flanagan, 2001, ** [77]	Mixed ICU, 64 pts	BAL Endotoxin	Clinical & BAL	Sens = 81%, spec = 87%, PPV = 67%, NPV = 95%.

AUC: area under the curve; ICU = intensive care unit; CRP = C-reactive protein; sens = sensitivity; spec = specificity; sTREM-1 = soluble triggering receptor expressed on myeloid cells; BAL = bronchoalveolar lavage; PCT = procalcitonin; EF = elastin fiber; NBLF = non-directed bronchial lavage fluids; EU = endotoxin units; *retrospective; **consecutive; ^#^convenient.

Procalcitonin (PCT) and C-reactive protein (CRP) measurements have been shown to improve the clinical accuracy in identifying patients with SIRS caused by infection from SIRS of other causes. Serum PCT levels had a better performance than alveolar PCT concentrations, with a sensitivity of 41% and a specificity of 100% [71]. In patients after cardiac arrest and return of spontaneous circulation, PCT had a sensitivity of 100% and a specificity of 75% for the diagnosis of VAP [72]. Póvoa *et al*. showed that CRP (>9.6 mg/dl) had a good accuracy for VAP, with sensitivity of 87% and a specificity of 88% in a population of general ICU patients [73]. In addition, in 47 patients with microbiologically confirmed VAP, high CRP levels were associated with poor outcome [74].

The detection of soluble triggering receptor expressed on myeloid cells (sTREM)-1 in BAL fluid may be useful in establishing or excluding a diagnosis of bacterial or fungal pneumonia. Gibot *et al*. [75] studied 148 patients with suspected pneumonia and measured sTREM-1 in their BAL fluid. These authors showed that the presence of sTREM by itself was more accurate than any clinical findings or laboratory values in identifying the presence of bacterial or fungal pneumonia. Questions about the lack of a gold standard definition for VAP may have hampered classification of some of patients. In 28 critically ill mechanically ventilated patients, Determann *et al*. [76] reported an increase in sTREM-1 levels in non-directed BAL fluid obtained from patients who developed VAP (*n *= 9) in contrast to those who did not. A cutoff value for BAL fluid sTREM-1 levels of 200 pg/ml had a sensitivity of 75% and specificity of 84% in diagnosing pneumonia.

The value of endotoxin measurements in BAL fluid was also investigated, as approximately 70% of cases of VAP are caused by Gram-negative bacteria. Flanagan *et al*. [77] reported an increased concentration of endotoxin in bronchoscopic BAL and non-directed BAL fluid of patients with VAP. An endotoxin concentration of 6 EU/ml yielded the optimal operating characteristics (sensitivity of 81% and specificity of 87%). In 40 samples of BAL fluid from patients with multiple trauma requiring prolonged mechanical ventilation, Pugin *et al*. [78] showed a relation between the concentration of endotoxin in lavage fluid and the quantity of Gram-negative bacteria. An endotoxin level greater than or equal to 6 EU/ml distinguished patients with Gram-negative bacterial pneumonia from colonized patients, and from those with pneumonia due to Gram-positive cocci.

The studies evaluating the value of the previously mentioned biomarkers are limited by the lack of a gold standard used to diagnosis VAP. EF and sTREM-1 were compared to clinical diagnosis *ad hoc *[75,76], PCT was compared to mini-BAL [71], and CRP to Johanson criteria [73].

In summary: CRP, PCT, and sTREM are promising biomarkers for diagnosing VAP, while EF and endotoxin concentrations are of limited value. Further studies are needed to fully determine the diagnostic accuracy of these and other biomarkers.

## Discussion

Evaluating the performance of various diagnostic tests in patients with suspected VAP is challenging. The absence of a good gold standard for comparison is the main limiting factor in assessing these tests. Establishing a diagnosis of VAP, based on pathology or histology plus culture of the lung tissue, has a considerable degree of uncertainty, however, it is considered the best available gold standard [8]. Pathologic examination of lung tissue is not a perfect gold standard because patients who die are not representative of all patients with VAP. Moreover, many patients die after some days of antibiotic administration, which may alter results of bacteriologic analysis. In addition, pathology or tissue cultures may include non-diseased lung tissue leading to false negative results, and diagnostic criteria based on pathologic examination of lung tissue are not well defined [27,79]. Fabregas *et al*. [79] found that the histology and microbiology of post-mortem lung biopsies were poorly correlated, challenging the value of histological examination of lung tissue in diagnosing VAP.

The etiology of VAP probably involves microaspiration of secretions accumulating above the cuff of endotracheal tube [80]. These foci of microinfection may remain localized causing local bronchiolitis without clinically relevant pneumonia or proceed to develop into micro- or macro-bronchopneumonia. The multifocal, heterogeneous nature of VAP is one of the reasons why it is so difficult to establish a diagnosis of VAP. Biopsy specimens can miss the area of active disease. Alternatively, positive results may represent an area of clinically silent early bronchiolitis or resolving bronchial pneumonia. Likewise, cultures can miss the area of active disease (yielding false-negative results) or can detect clinically benign areas of bacterial colonization (yielding false positive results). In addition, the many antibiotics given to critically ill patients may decrease the diagnostic yield of bacterial cultures [24,79].

Associated cardiorespiratory comorbidities are another source of bias in diagnosing VAP. Autopsies of ventilated patients with suspected pneumonia frequently reveal a substantial burden of alternative or coexisting pulmonary diseases that can also cause fever, impaired gas exchange, increased secretions, and radiographic opacities. These other conditions include thromboembolic disease, hemorrhage, diffuse alveolar damage, fibrosis, atelectasia, carcinoma, lymphoma, and others [11,12,24,27,28]. The high prevalence of coexisting pulmonary diseases in ventilated patients further complicates attempts to clinically diagnose VAP.

The major limitation of the clinical approach to diagnosis is that it consistently leads to more antibiotic therapy than when therapy decisions are based on the findings of invasive lower respiratory tract samples. The clinical approach is overly sensitive, and patients can be treated for pneumonia when another non-infectious process is responsible for the clinical findings.

**Figure 1:**
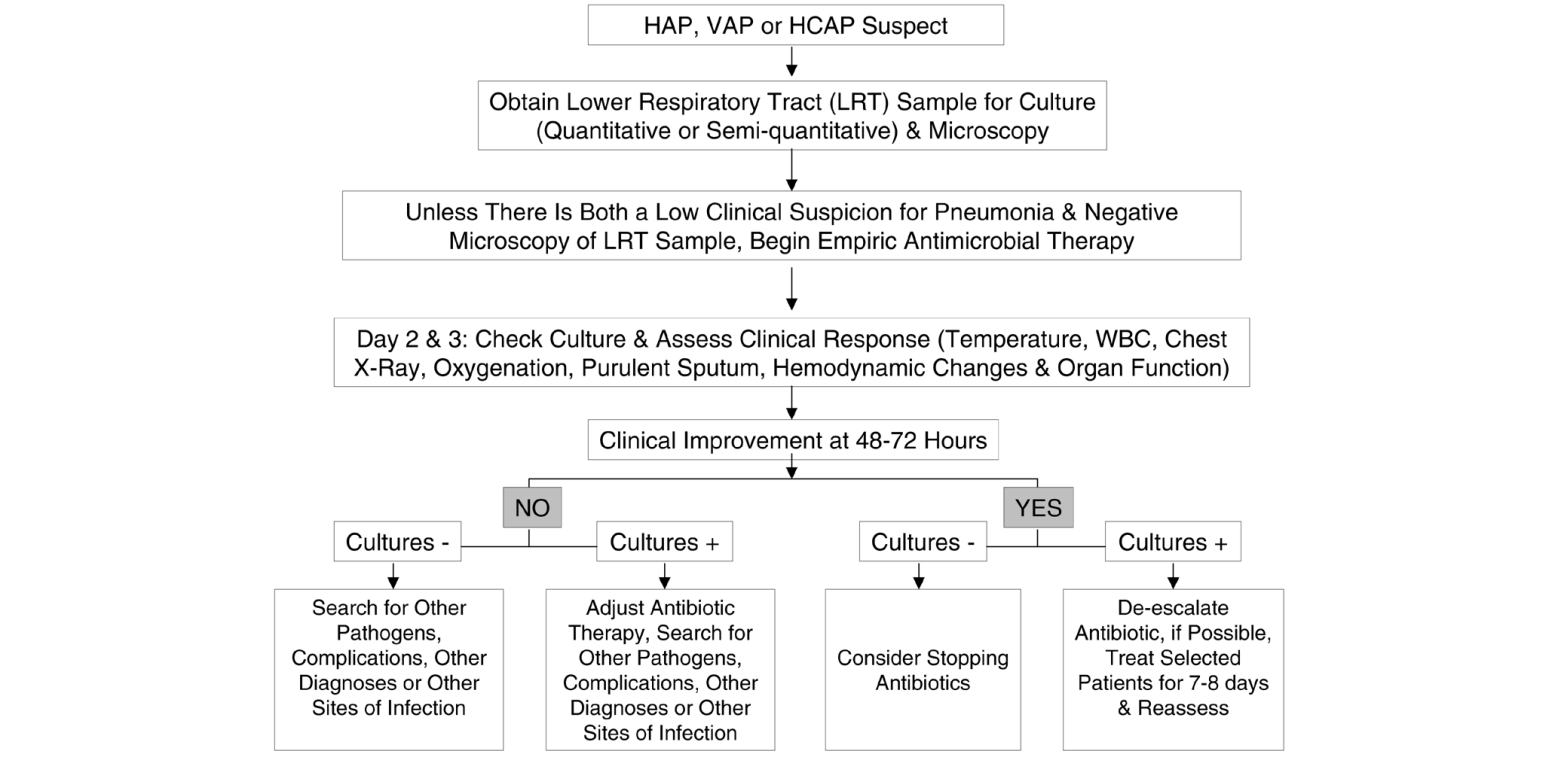
Summary of the management strategies for a patient with suspected hospital-acquired pneumonia (HAP), ventilator-associated pneumonia (VAP), or healthcare-associated pneumonia (HCAP). The decision about antibiotic discontinuation may differ depending on the type of sample collected (PSB, BAL, or endotracheal aspirate), and whether the results are reported in quantitative or semiquantitative terms. From [1] (with permission).

As none of the available diagnostic tests, performed alone, can provide an accurate diagnosis of VAP, a diagnostic strategy incorporating several criteria seems to be a good compromise. On the basis of clinical data, patients with clinical suspicion of VAP should be further evaluated by imaging procedures, bacteriological cultures, and biomarkers (Figure 1). The results of complementary diagnostic procedures should be used to refine the probability of diagnosing VAP and guide therapeutic decisions. Quantitative cultures should be performed on endotracheal aspirates or samples collected bronchoscopically, each technique having its own methodological limitations. Delays in the initiation of adequate antibiotic therapy increase mortality of VAP and thus therapy should not be postponed for the purpose of performing diagnostic studies in patients who are clinically unstable. The presence of organ dysfunction may necessitate the prompt initiation of antibiotic therapy. Garrard and A'Court [81] recommended regular, repeated surveillance with a simple, inexpensive, and well tolerated lavage technique in addition to daily clinical scoring to identify patients who may have VAP. A recent meta-analysis [82] of four randomized studies with a total of 628 patients showed that invasive strategies for the diagnosis of VAP did not alter mortality. In a recent multicenter trial, Heyland *et al*. [83] randomized 740 patients who were receiving mechanical ventilation and who had suspected VAP after 4 days in the ICU to undergo either BAL with quantitative culture of the BAL fluid or endotracheal aspiration with non-quantitative culture of the aspirate. Empirical antibiotic therapy was initiated in all patients until culture results were available, at which point a protocol of targeted therapy was used for discontinuing or reducing the dose or number of antibiotics, or for resuming antibiotic therapy to treat a pre-enrollment condition if the culture was negative. There was no significant difference in outcomes or the use of antibiotics. The most likely explanation for this lack of effect on outcome is that prompt adequate initial antimicrobial coverage is the crucial issue affecting survival. Inappropriate or inadequate treatment refers to the use of antibiotics with either limited or no in vitro activity against the microorganism causing the infection. Since invasive sampling for suspected VAP does not directly affect the initial antibiotic prescription, it is not surprising that it does not alter mortality. Because of the nature of the technology, the culture results from bronchoscopy become available only after the crucial period when the clinician can intervene to maximal effect [82].

## Conclusion

Clinical criteria, used in combination, may be helpful in diagnosing VAP; however, the considerable inter-observer variability and the moderate performance should be taken into account. Bacteriologic data do not increase the accuracy of diagnosis as compared to clinical diagnosis. Quantitative cultures obtained by different methods, including BAL, pBAL, PSB or TBA seem to be rather equivalent in diagnosing VAP. Blood cultures are relatively insensitive to diagnose pneumonia. The rapid availability of cytological data, including inflammatory cells and Gram stains, may be useful in initial therapeutic decisions in patients with suspected VAP. CRP, PCT, and sTREM are promising biomarkers in diagnosing VAP. An integrated approach should be followed in diagnosing and treating patients with VAP, including early antibiotic therapy and subsequent rectification according to clinical response and results of bacteriologic cultures.

## Key messages

• Clinical criteria, used in combination, may be helpful in diagnosing VAP, however, the considerable inter-observer variability and the moderate performance should be taken in account.

• Bacteriologic data do not increase the accuracy of diagnosis as compared to clinical diagnosis. Quantitative cultures obtained by different methods, including BAL, pBAL, PSB or TBA seem to be rather equivalent in diagnosing VAP.

• The rapid availability of cytological data, including inflammatory cells and Gram stains, may be useful in initial therapeutic decisions in patients with suspected VAP.

• CRP, PCT, and sTREM are promising biomarkers in diagnosing VAP.

• An integrated approach should be followed in diagnosing and treating patients with VAP, including early antibiotic therapy and subsequent rectification according to clinical response and results of bacteriologic cultures.

## Abbreviations

ARDS = Acute respiratory distress syndrome; BAL = Bronchoalveolar lavage; CFU = Colony forming units; CPIS = Clinical pulmonary infection score; CRP = C-reactive protein; EF = Elastin fiber; ICU = Intensive care unit; NNIS = National Nosocomial Infection Surveillance; pBAL = Protected bronchoalvolar lavage; PCT = Procalcitonin; PSB = Protected specimen brush; SIRS = Systemic inflammatory response syndrome; sTREM = Soluble triggering receptor expressed on myeloid cells; TBA = Tracheobronchial aspirate; VAP = Ventilator-associated pneumonia.

## Competing interests

KR and FB have received fees from BRAHMS AG for speaking and for scientific advice. AR, NY, FT, and YS declare that they have no competing interests.

## Authors' contributions

All authors participated in the design of the study. AR, NY, and FT contributed to data collection. AR, NY and YS drafted the manuscript. MR, KR and FB revised the article. All authors read and approved the final manuscript.
